# Viral shedding and environmental dispersion of two clade 2.3.4.4b H5 high pathogenicity avian influenza viruses in experimentally infected mule ducks: implications for environmental sampling

**DOI:** 10.1186/s13567-024-01357-z

**Published:** 2024-08-12

**Authors:** Fabien Filaire, Kateri Bertran, Nicolas Gaide, Rosa Valle, Aurélie Secula, Albert Perlas, Charlotte Foret-Lucas, Miquel Nofrarías, Guillermo Cantero, Guillaume Croville, Natàlia Majó, Jean-Luc Guerin

**Affiliations:** 1 IHAP, Université de Toulouse, INRAE, ENVT, Toulouse, France; 2 LanXess Group, THESEO France, Lanxess Biosecurity, Laval, France; 3 Unitat Mixta d’Investigació IRTA-UAB en Sanitat Animal, Centre de Recerca en Sanitat Animal (CReSA), Campus de la Universitat Autònoma de Barcelona (UAB), 08193 Bellaterra, Catalonia Spain; 4 Programa de Sanitat Animal, IRTA, Centre de Recerca en Sanitat Animal (CReSA), Campus de la Universitat Autònoma de Barcelona (UAB), 08193 Bellaterra, Catalonia Spain; 5 Departament de Sanitat i Anatomia Animals, Facultat de Veterinària, Campus de la Universitat Autònoma de Barcelona (UAB), 08193 Bellaterra, Catalonia Spain

**Keywords:** Viral shedding, HPAIV, aerosol, dust, environment, surveillance

## Abstract

High pathogenicity avian influenza viruses (HPAIVs) have caused major epizootics in recent years, with devastating consequences for poultry and wildlife worldwide. Domestic and wild ducks can be highly susceptible to HPAIVs, and infection leads to efficient viral replication and massive shedding (i.e., high titres for an extended time), contributing to widespread viral dissemination. Importantly, ducks are known to shed high amounts of virus in the earliest phase of infection, but the dynamics and impact of environmental contamination on the epidemiology of HPAIV outbreaks are poorly understood. In this study, we monitored mule ducks experimentally infected with two H5N8 clade 2.3.4.4b goose/Guangdong HPAIVs sampled in France in 2016–2017 and 2020–2021 epizootics. We investigated viral shedding dynamics in the oropharynx, cloaca, conjunctiva, and feathers; bird-to-bird viral transmission; and the role of the environment in viral spread and as a source of samples for early detection and surveillance. Our findings showed that viral shedding started before the onset of clinical signs, i.e., as early as 1 day post-inoculation (dpi) or post-contact exposure, peaked at 4 dpi, and lasted for up to 14 dpi. The detection of viral RNA in aerosols, dust, and water samples mirrored viral shedding dynamics, and viral isolation from these environmental samples was successful throughout the experiment. Our results confirm that mule ducks can shed high HPAIV titres through the four excretion routes tested (oropharyngeal, cloacal, conjunctival, and feather) while being asymptomatic and that environmental sampling could be a non-invasive tool for early viral RNA detection in HPAIV-infected farms.

**Supplementary Information:**

The online version contains supplementary material available at 10.1186/s13567-024-01357-z.

## Introduction

High pathogenicity avian influenza virus (HPAIV) has become a major threat for the poultry industry and wildlife biodiversity since the worldwide spread of the emerging clade 2.3.4.4b derived from the A/goose/Guangdong/1/1996 (Gs/GD) H5 lineage [1–3]. These viruses affect a remarkably wide range of bird species, which could explain the massive viral dissemination in wild bird populations globally [1, 4]. Among poultry species, waterfowl are more susceptible to clade 2.3.4.4b viruses than are gallinaceous birds [5–7]. Ducks were found to have lower mean bird infectious and lethal doses than chickens when infected with clade 2.3.4.4b virus but still exhibited high viral shedding during the course of the infection [7]. These features, which are typically associated with mild to low clinical signs upon infection, play a crucial role in the epidemiology of HPAI in duck farming [5, 6, 8, 9]. Once infected birds shed the virus, the environment plays a major role in viral persistence and viral spread; both airborne and waterborne viral transmission are thought to drive intra- and inter-flock dissemination [10–12]. Since the virus is present in the environment, environmental sampling could be an alternative to bird swabbing for HPAIV monitoring since it is non-invasive for birds, less time-consuming, and does not require the intervention of trained staff. Field investigations by our group suggested that the collection of aerosols, or even dust, allows for the detection of high viral loads, even early in the course of infection at the flock level [13].

Here, we investigated the kinetics of viral shedding and environmental contamination in ducks experimentally infected with clade 2.3.4.4b H5N8 HPAIVs. We used two different strains isolated from French outbreaks in 2017 and 2020 to evaluate potential changes in their biological properties. Our objective was to assess the potential application of environmental sampling and the impact of the persistence and dissemination of virus particles in the environment of poultry houses.

## Materials and methods

### Viruses

Two clade 2.3.4.4b Gs/GD lineage H5N8 HPAIV isolates were used as challenge viruses: A/mulard duck/France/171201 g/2017 (H5N8) HPAIV (H5N8/2017) reverse genetics-engineered (accession numbers MK859904 to MK859911) [14] and A/Mule_duck/France/20353/2020 (H5N8/2020) (accession numbers MZ166297 to MZ166304). H5N8/2020 was obtained from pooled feather samples from an infected duck farm in France during the 2020–2021 epizootic. Both viruses were propagated and titrated by allantoic sac inoculation in 9- to 11-day-old specific-pathogen-free (SPF) embryonated chicken eggs by standard methods [15].

### Animals and housing

Thirty-two 3-week-old mule ducks (*Cairina moschata* × *Anas platyrhynchos*) were obtained from a commercial producer (courtesy of Manel Vinyes, GALLSA, Tarragona, Spain). Birds were randomly allocated into two rooms in the animal BSL-3 facilities. The birds had ad libitum access to feed and water. Each room was provided with a swimming pool 1.5 m in diameter and 30 cm in depth with an access ramp. Oropharyngeal (OP) and cloacal (CL) swabs and blood samples were collected from all the birds prior to inoculation. Current infection was tested by qRT-PCR in swab samples [16], and the presence of antibodies from previous exposure was determined by competitive ELISA (AI MultiS-Screen Ab Test, IDEXX) and hemagglutination inhibition (HI) assays [15].

### Experimental design, clinical monitoring, and biological sampling

After 1 week of acclimation, 10 birds per room were intrachoanally inoculated with 10^5^ mean embryo infectious doses of either H5N8/2017 or H5N8/2020. To evaluate viral transmission, six non-inoculated ducks were added to each room at 1 day post-inoculation (dpi). Clinical signs were monitored daily for 14 dpi.

The following clinical scoring system was used [17]: 0 (healthy), 1 (sick with one HPAIV typical clinical sign), 2 (severely sick with two or more HPAIV typical clinical signs), and 3 (dead). Severely sick birds were euthanized by intravenous overdose of sodium pentobarbital (140 mg/kg) under intravenous anaesthesia with ketamine/xylazine (201 mg/kg) and counted as dead the next day. At 14 dpi, the surviving birds were bled and euthanized. To investigate individual viral shedding, oropharyngeal (OP), cloacal (CL), and conjunctival (CJ) swabs, as well as feather pulp (FP) samples, were collected from inoculated and contact birds at 2, 4, 7, 10, and 14 dpi. For the CJ samples, the conjunctival mucosa of the birds was gently swabbed. Immature wing or caudal feathers were sampled for FP extraction.

### Serology

Sera from all surviving birds collected at 14 dpi were tested by a commercial competitive ELISA (Innovative Diagnostics, Grabel, France) to evaluate seroconversion and by an HI assay to detect antibody levels. The HI assay was performed using standard methods and homologous antigens [15].

### Environmental sampling

To evaluate viral contamination in the environment from experimentally inoculated and contact ducks, aerosol, dust, and water samples were collected. Aerosol sampling was performed using two dry cyclonic air samplers: the Coriolis Compact (Bertin Technologies, Montigny-le-Bretonneux, France) and the National Institute of Occupational Safety and Health (NIOSH) BC 251. Briefly, the Coriolis compact sampler, with a 50 L/min calibrated flow rate, enables the dry collection of all aerosol particles ranging from 500 nm to 10 µm in size. The 2-stage bioaerosol cyclone (BC) NIOSH BC 251 sampler, with a calibrated 3.5 L/min flow rate, enables dry collection and sorting of the aerosol particles into > 4 µm, 1–4 µm, and < 1 µm fractions. Thus, aerosols are separated into a 15 mL tube for the largest fraction, a 1.5 mL tube for the medium fraction, and a 37 mm diameter polytetrafluoroethylene (PTFE) filter with a 1.5 µm pore for the smallest fraction. Both instruments were used simultaneously at every sampling time point—20 min for the Coriolis Compact and 40 min to 1 h for the NIOSH BC 251—at approximately 1 m above ground and 2 m away from each other to avoid air flow interference.

Dust samples were collected using dry gauzes, one on the fences and one on the feeders, which were immediately placed in individual sealable bags. The aim was to collect the maximum amount of dust from these surfaces while avoiding contamination with food and feces, which could inhibit molecular analysis.

Pool water samples and drinking water samples were collected using 50 mL Falcon tubes. While the pool water was only refilled when needed and not renewed at any time point during the experiment, the drinking water was changed daily after the sampling.

All environmental sampling was performed in each group at 1, 2, 3, 4, 5, 7, 10, and 14 dpi. Additionally, aerosol sampling with the Coriolis Compact was performed before challenge as a negative control. All samples were stored at 4 °C for up to 2 days post-sampling and at -80 °C until processing.

**Figure 1:**
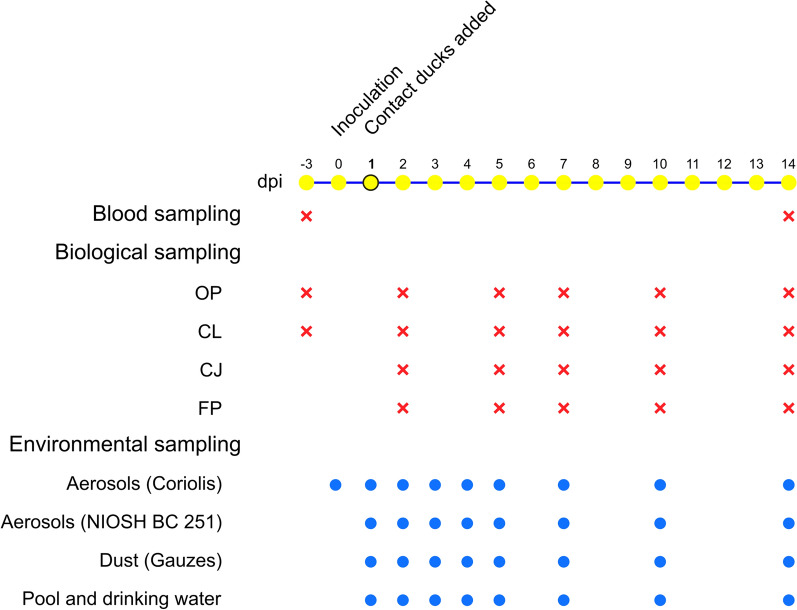
**General overview of the experimental design.** Blood and biological sampling were performed on all living birds on the day of the sampling. Aerosols were collected using the Coriolis Compact (Bertin Technologies) and the National Institute of Occupational Safety and Health (NIOSH) BC 251. Dust was independently sampled on the walls and feeders using gauzes. Pool water and drinking water were collected using 50 mL Falcon tubes. *OP* oropharyngeal swabs, *CL* cloacal swabs, *CJ* conjunctival swabs, *FP* feather pulp, *dpi* days post-inoculation.

An overview of the experimental design is summarized in Figure 1.

### Processing methods

Before RNA extraction, all the samples were prepared as follows. The swabs (OP, CL, and CJ) and FPs were individually placed into single 1.5 mL centrifuge tubes filled with 500 µL of 1X phosphate buffered saline (PBS) and vigorously vortexed for 10–15 s.

Aerosols collected with the Coriolis Compact sampler and the two largest particle sizes of the NIOSH BC 251 sampler were resuspended in 1 mL of PBS. The collection tubes were vigorously vortexed for 5–15 s to remove particles from the tube walls and edges. When needed, up-and-down pipetting was additionally performed to detach the particles from the tube’s edges. The NIOSH BC 251 third fraction membrane filter was carefully removed from the cassette and placed into a dry 50 mL collection tube. The filter was dry vortexed for 10–15 s before adding 1.5 mL of PBS and then vortexed again. The aerosol liquid resuspension was aliquoted into a 1.5 mL centrifuge tube. Gauze with dust was processed by adding 20 mL of PBS to sealable bags, hand-massaging the contents for 2–3 min, and collecting and aliquoting the supernatants into 1.5 mL centrifuge tubes. Pool and drinking water samples were aliquoted into 2 mL centrifuge tubes.

All samples were stored at −80 °C at IRTA-CReSA for the duration of the experiment and then shipped to the National Veterinary School of Toulouse BSL-3 for analysis.

### Viral RNA detection

Total RNA from all samples (bird samples and environmental samples individually) was extracted using the magnetic bead-based ID Gene Mag Fast Extraction Kit (Innovative Diagnostics, Grabel, France) associated with the KingFisher 96 automated magnetic extraction robot (Thermo Fisher, Carlsbad, CA, USA) following the manufacturer’s instructions. The presence of AIV RNA from the H5 subtype was detected by performing 1-step real-time reverse transcription quantitative PCR (rRT-qPCR) using an influenza H5/H7 Triplex kit (Innovative Diagnostics, Grabel, France) (Additional file 1). The rRT-qPCR amplification procedure consisted of 40 cycles. Unamplified vRNA was considered negative.

### Virus isolation

To assess the extent and duration of HPAIV environmental spread, the presence of infectious particles was determined in RNA-positive aerosol, water, and dust samples. Both drinking water and pool water were used for viral isolation. For aerosols and dust, samples with higher RNA yields were selected. Therefore, Coriolis aerosols and wall dust samples were selected for viral isolation from aerosols and dust, respectively.

Virus isolation was conducted in 9- to 11-day-old SPF embryonated chicken eggs. Briefly, 200 µL of each sample was mixed with 400 µL of penicillin (1000 U/mL) and streptomycin (1 mg/mL). Three eggs per sample were inoculated with 150 µL of the solution and kept in a humidity chamber at 37 °C for 48 h before being placed at 4 °C for 12 h. Allantoic fluid was collected from each egg and directly analysed via RT-qPCR targeting the H5 subtype (Influenza H5/H7 Triplex kit, Innovative Diagnostics, France) and a hemagglutination assay [15]. Up to three successive passages per sample were performed.

### Data analysis

All analyses were performed using R Statistical Software version 4.1.1 [18]. Statistical analyses were performed using the nlme package [19]. Figures were made using the ggplot2 package [20].

## Results

### Clinical signs and mortality

**Figure 2:**
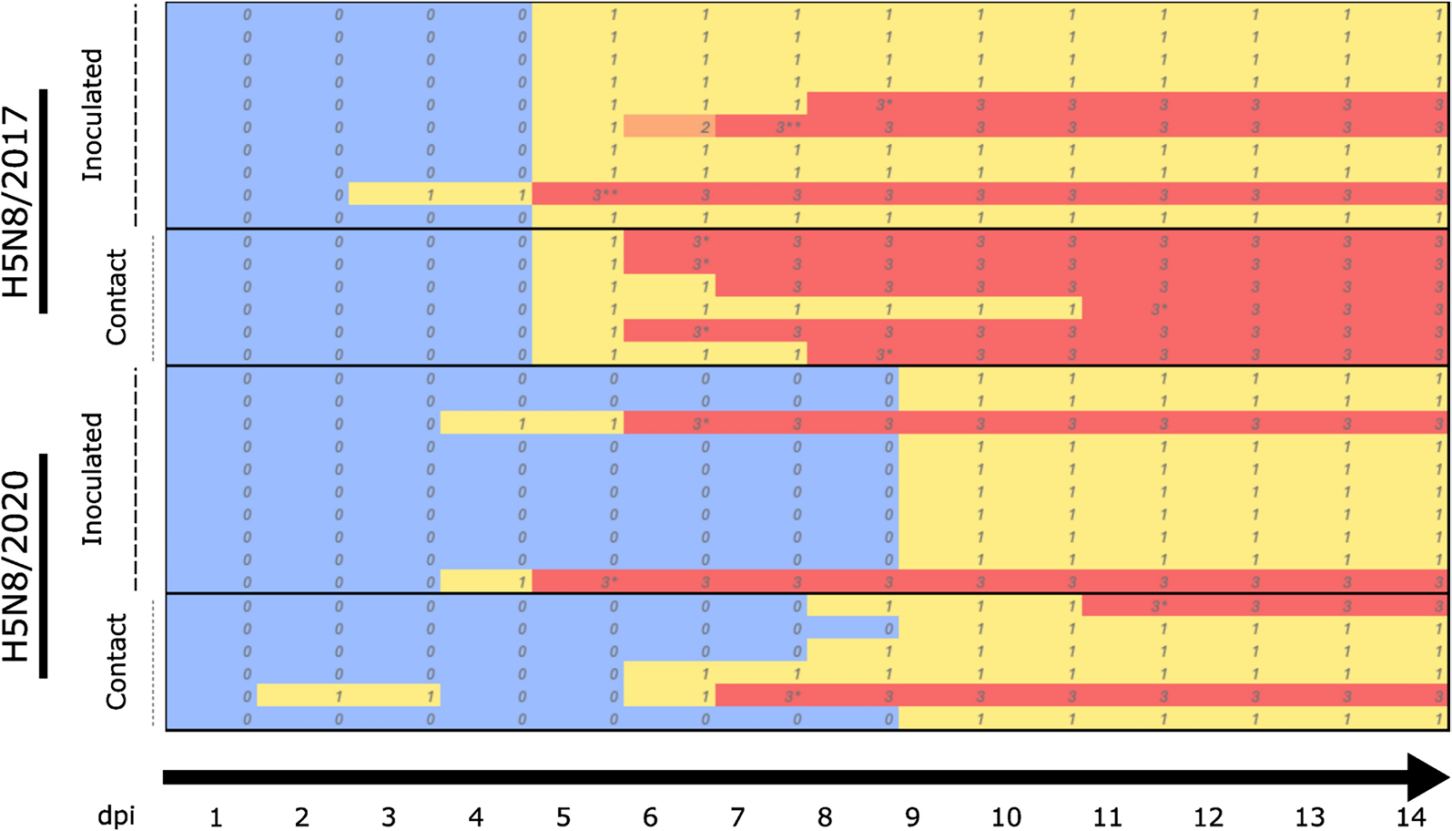
**Evolution of clinical scoring over time for ducks experimentally infected with H5N8/2017 and H5N8/2020 HPAIVs**. The H5N8/2017 and H5N8/2020 HPAIVs correspond to A/mulard duck/France/171201 g/2017 (H5N8) and A/Mule_duck/France/20353/2020, respectively. Each row represents a single bird. Birds were grouped based on the virus strain and exposure route (inoculated vs contact). 0: healthy (green), 1: sick with one HPAIV typical clinical sign (yellow), 2: severely sick with two or more HPAIV typical clinical signs (orange), 3: dead bird (found death or euthanasia) (red). dpi: days post-inoculation. *: severely sick birds euthanized for ethical reasons, **: birds that were found dead.

**Figure 3:**
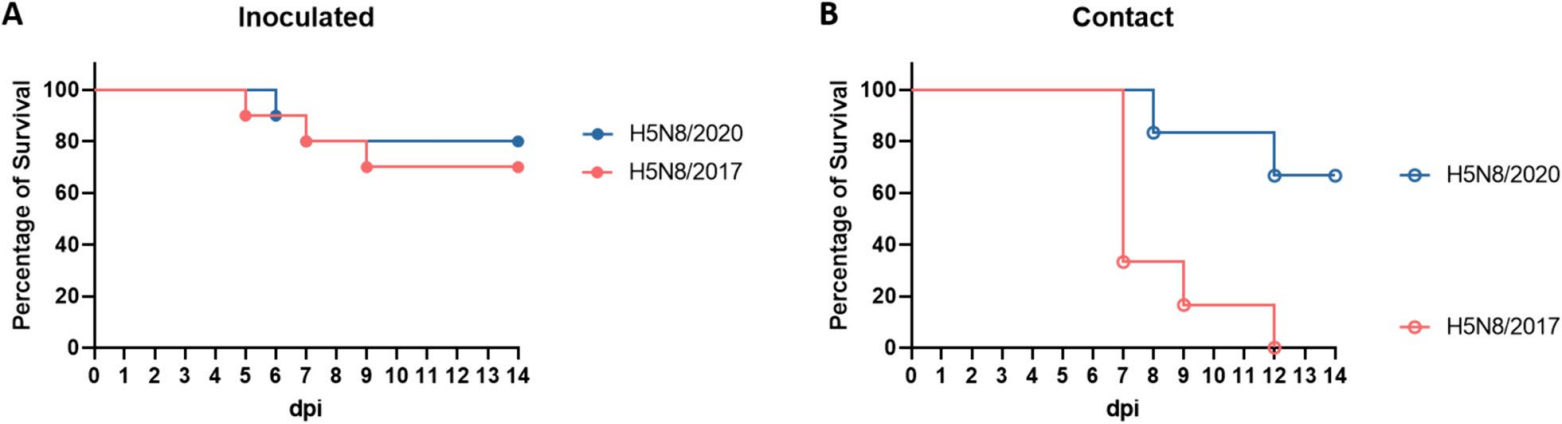
**Evolution of the survival rate of ducks experimentally infected with H5N8/2017 and H5N8/2020 HPAIVs**. The H5N8/2017 and H5N8/2020 HPAIVs correspond to A/mulard duck/France/171201 g/2017 (H5N8) and A/Mule_duck/France/20353/2020, respectively. **A** Percentage of survival in inoculated ducks. **B** Percentage of survival in contact ducks. *dpi* days post-inoculation.

All the acclimated birds were confirmed to be AIV PCR negative and AIV serologically negative by both HI and ELISA and were clinically healthy before challenge. Clinical signs started at 3 dpi and 4 dpi for H5/2017- and H5/2020-inoculated birds, respectively. Clinical signs included non-specific depression to prostration and inability to stand upright, neurological signs (ataxia, head tremor, head tilt), and unresponsiveness to visual stimuli. For inoculated birds, mortality started at 5 and 6 dpi for the H5N8/2017- and H5N8/2020-inoculated groups, respectively, and lasted for 4 and 2 days, respectively (Figures 2 and 3). Overall, the mortality rates of inoculated birds were 30% (H5N8/2017) and 20% (H5N8/2020), with mean death times (MDTs) of 7 days (H5N8/2017) and 6.5 days (H5N8/2020), respectively (Figure 2). The survival rate, analysed by the chi-square test, was not significantly different between the groups (*p* > 0.05). For contact birds, mortality started at 7 dpi (i.e., 6 days post-contact exposure) with H5N8/2017 and 8 dpi (i.e., 7 days post-contact exposure) with H5N8/2020, and lasted for 5 days. The survival rate of contact birds was significantly greater in the H5N8/2020 group (*p* < 0.05). Overall, the mortality rates of contact birds were 100% (H5N8/2017) and 33% (H5N8/2020), with MDTs of 8.2 days (H5N8/2017) and 10 days (H5N8/2020), respectively (Figure 2). All inoculated and contact surviving birds seroconverted following challenge, as confirmed by HI and ELISA. The antibody titres included log_2_ 8.4 geometrical mean titres (GMTs) (H5N8/2017 inoculated ducks), log_2_ 8.6 GMTs (H5N8/2020 inoculated ducks), and log_2_ 7.5 GMTs (H5N8/2020 contact ducks) (Additional file 2).

### Viral excretion in ducks

**Figure 4:**
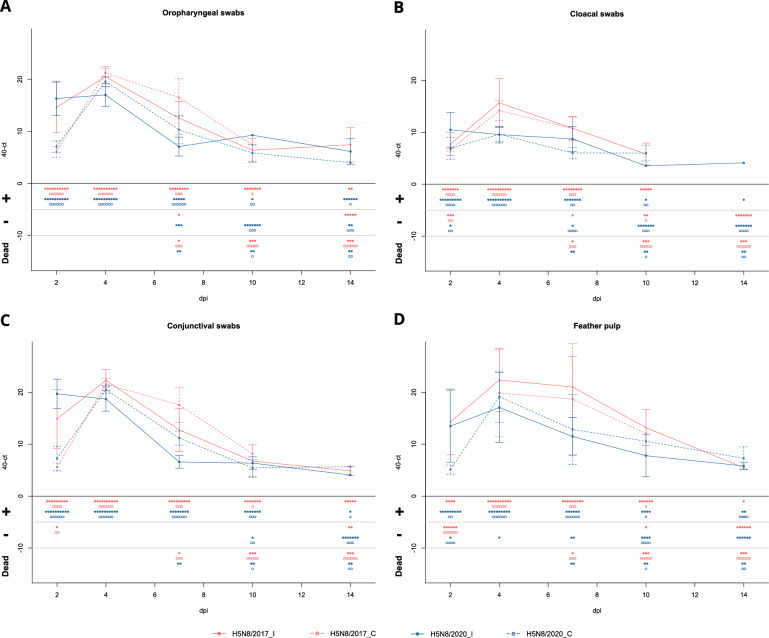
**Viral shedding in oropharyngeal, cloacal, and conjunctival swabs and feather pulp samples with animal status information**. Graph: dots and whiskers represent the mean amount of viral RNA detected and the standard deviation, respectively. Animal status: Each dot represents one bird. The health status of each bird is described in each panel. +  vRNA positive detection, −: vRNA negative detection, *dpi* days post-inoculation, *H5N8/2017_I* H5N8/2017 inoculated birds, *H5N8/2017_C* H5N8/2017 contact birds, *H5N8/2020_I* H5N8/2020 inoculated birds, *H5N8/2020_C* H5N8/2020 contact birds.

**Figure 5:**
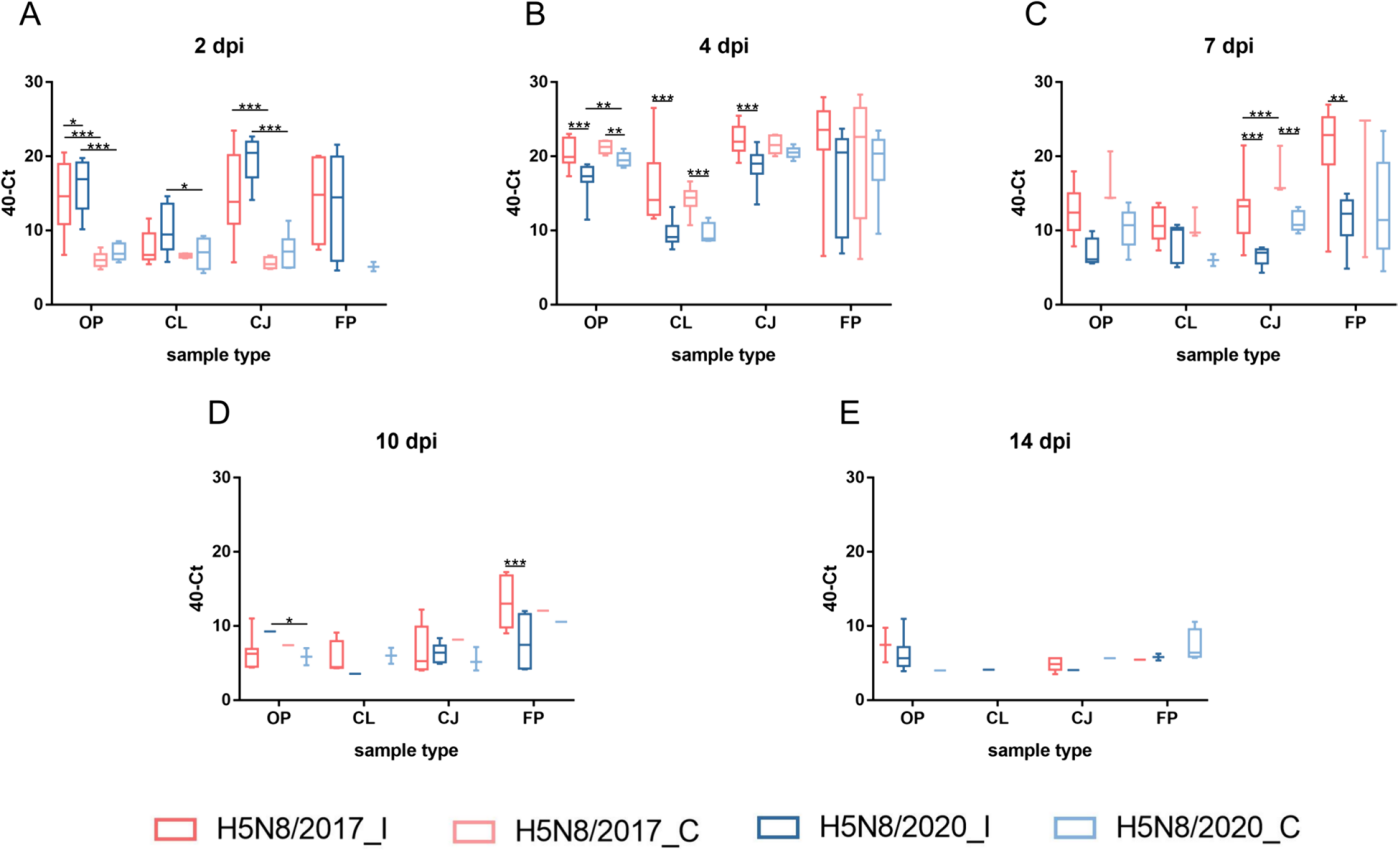
**Viral shedding in biological samples at each sampling time point.** *OP* oropharyngeal swabs, *CL* cloacal swabs, *CJ* conjunctival swabs, *FP* feather pulp, *dpi* days post-inoculation, *H5N8/2017_I* H5N8/2017 inoculated birds, *H5N8/2017_C* H5N8/2017 contact birds, *H5N8/2020_I* H5N8/2020 inoculated birds, *H5N8/2020_C* H5N8/2020 contact birds, *P < 0.05, **P < 0.01, ***P < 0.001, ****P < 0.0001. Statistical analysis: linear mixed ANOVA.

The overall profiles of excretion were similar regardless of the virus and infection route (inoculation vs contact) (Figure 4). In particular, viral RNA (vRNA) was first detected at 2 dpi, and viral shedding peaked at 4 dpi and gradually decreased until 10 dpi, when it stabilized until 14 dpi (Figures 4 and 5).

Viral RNA of both viruses was already detectable in OP swabs, CL swabs, CJ swabs, and FP samples at 2 dpi, in inoculated birds and in contact birds except for the H5N8/2017 FP samples (Figures 1, 4, and 5). At 2 dpi, the vRNA mean load was greater in the H5N8/2020 samples than in the H5N8/2017 samples (Figures 4 and 5A) for all sample types except for the FP samples, with significant differences in the OP swabs (Figure 5A). However, from 4 dpi onwards, the H5N8/2017 vRNA load was greater than that of H5N8/2020 in most samples and in both inoculated and contact birds (Figures 4 and 5). The H5N8/2020 vRNA load in FP and CL swabs was greater in contact birds than in inoculated birds from 4 to 14 dpi. This was also true for OP swabs, but only at 4 dpi and 7 dpi. For the H5N8/2017 samples, the vRNA load in OP and CJ swabs from contact birds was greater than that in inoculated birds at 4 and 7 dpi. From 10 dpi to the end of the experiment, FP, OP, and CJ samples enabled the detection of vRNA in more birds than CL swabs. The differences in Ct values between viruses may vary greatly, up to 10 Ct for feather pulp samples at 7 dpi.

### Viral RNA detection in the environment

**Figure 6:**
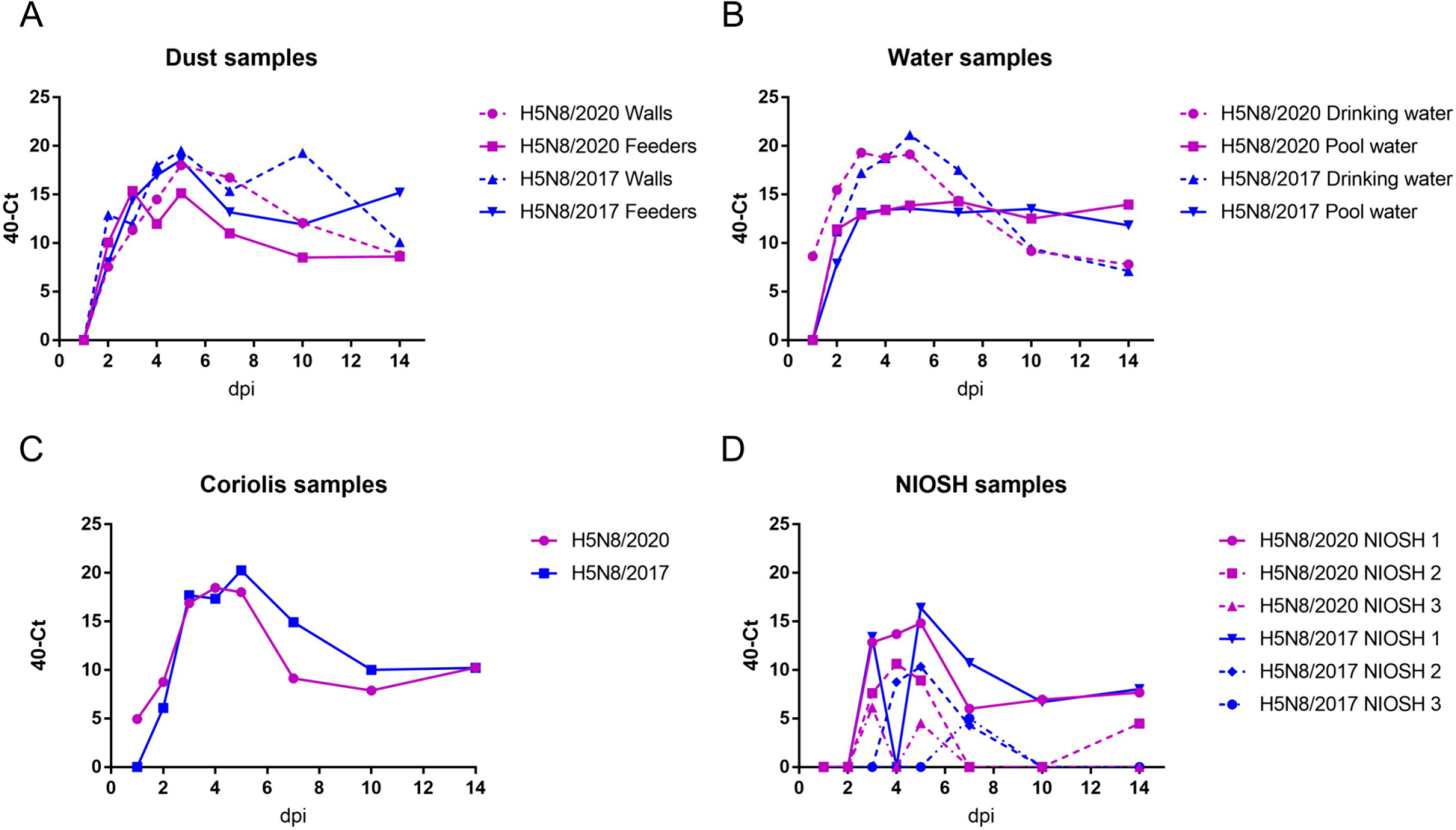
**Viral shedding monitoring in environmental samples. A** Dust samples collected using gauzes on walls and feeders separately. **B** Water samples collected from the drinking tank and the pool. **C** aerosols collected using the Coriolis Compact (Bertin Technologies, Montigny-le-Bretonneux, France). **D** Aerosols collected using the NIOSH BC 251. The NIOSH BC 251 model separates aerosols based on their size: NIOSH 1 < 4 µm, NIOSH 2 1-to-4 µm, and NIOSH 3 < 1 µm. dpi: days postinoculation.

To investigate bird-to-bird transmission, viral shedding, and the role of the environment in the spread of the virus, dust, water (drinking and pool), and aerosol samples were collected at different time points and analysed by rRT-qPCR targeting the H5 subtype. The results are presented in Figure 6.

Viral RNA was first detected in environmental samples as early as 1 dpi in the H5N8/2020 Coriolis aerosol sample (C_t_: 35.05) and drinking water sample (C_t_: 31.37) and at 2 dpi in the H5N8/2017 samples. Overall, the kinetics for all the environmental samples were similar regardless of the virus. In particular, the vRNA load gradually increased and peaked at 5 dpi before decreasing until 10 dpi. From 10 to 14 dpi, the environmental vRNA load remained roughly stable. Dust samples from walls yielded higher vRNA loads than dust from feeders for both viruses (Figure 6A). This kinetic trend was different for the pool water samples; for both viruses, the vRNA load peaked at 3 dpi (H5N8/2017 average C_t_: 26.9, sd: 0.65; H5N8/2020 average C_t_: 26.81, sd: 0.67) and remained stable until the end of the experiment (Figure 6B, Additional files 3 and 4). Viral RNA detection in aerosol NIOSH samples had similar kinetics to that in Coriolis samples, even though the first positive samples were not detected before 3 dpi. Interestingly, for both viruses, the vRNA load decreased with increasing fraction size.

### Virus isolation from environmental samples

To investigate the presence of infectious particles in the environment and their association with viral shedding and clinical signs, virus isolation from SPF chicken embryonated eggs was performed from a selection of PCR-positive samples and time points, i.e., aerosols (Coriolis), dust (walls), pool water, and drinking water (Table 1).

**Table 1:** **Virus isolation in embryonated chicken eggs from environmental samples**

Viral strain	Sample	1 dpi	2 dpi	3 dpi	5 dpi	10 dpi	14 dpi
H5N8/2017	Aerosols (Coriolis)	ND	−	−	−	−	+
	Dust (walls)	ND	−	−	+	−	−
	Pool water	ND	−	−	+	−	−
	Drinking water	−	+	ND	+	−	−
H5N8/2020	Aerosols (Coriolis)	−	+	ND	+	−	−
	Dust (walls)	ND	+	ND	+	ND	+
	Pool water	ND	+	ND	+	−	−
	Drinking water	+	+	ND	+	ND	+

*dpi* days post-inoculation, *ND* not done, + positive, − negative. Viral shedding from birds assessed by oropharyngeal and cloacal swabs started at 2 dpi for both viruses.

For the H5N8/2017 virus, very few PCR-positive samples yielded infectious viruses (Figure 6, Table 1 and Additional file 3). The earliest recovery of the infectious virus from the drinking water occurred at 2 dpi. At 5 dpi, infectious particles could be isolated from dust, pool water, and drinking water. Coriolis aerosol samples only enabled virus isolation at 14 dpi, the only positive sample at this time point (Table 1).

In contrast to H5N8/2017 samples, H5N8/2020 environmental samples were successfully isolated from drinking water at 1 dpi and from all sample types at 2 dpi and 5 dpi. Infectious particles were also isolated from wall dust and drinking water at 14 dpi (Table 1).

## Discussion

Domestic and wild waterfowl play a crucial role in the worldwide spread of HPAIVs of the Gs/GD lineage due to their high susceptibility and efficient viral shedding [5, 6, 8, 9, 21, 22]. Wild and domestic ducks are key players at the wild-domestic interface [10, 22, 23] and in maintaining these viruses in the environment [5, 6, 24]. By monitoring H5N8/2017 and H5N8/2020 clade 2.3.4.4b Gs/GD HPAIV-infected mule ducks in an experimental setting, we investigated viral shedding dynamics, bird-to-bird transmission, the role of the environment as transmission vehicle, and the reliability of environmental sampling for viral detection.

Clinical signs were first observed at 3 dpi and 4 dpi in H5N8/2017- and H5N8/2020-inoculated ducks, respectively, and at 5 dpi and 6 dpi in H5N8/2017- and H5N8/2020-contact ducks, respectively. These observations indicate a 3-to-5-day presymptomatic period. This presymptomatic period is in agreement with previous experimental infections using different HPAIVs in ducks [25–27] and with mathematical modelling approaches performed on field data from 2016–2017 and 2020–2021 clade 2.3.4.4b HPAIV outbreaks [28, 29]. Lambert et al. calculated that during the H5N8 HPAIV 2020–2021 epizootic in France, the interval between the onset of clinical signs on two close farms was, on average, 4.78 days [28], suggesting that farm-to-farm transmission occurs during the presymptomatic period.

The mortality rates obtained here (30%, H5N8/2017 inoculated; 20%, H5N8/2020 inoculated; 100%, H5N8/2017 contact; and 33%, H5N8/2020 contact) confirmed previous results using clade 2.3.4.4a and clade 2.3.4.4b viruses in experimentally infected domestic birds [21]. However, numerous studies performed on different duck species have not shown any mortality in either inoculated or contact birds infected with recent clade 2.3.4.4a or clade 2.3.4.4b viruses [12, 23, 27]. Differences in mortality rates among different studies could be explained by the viral strain, the age of the birds, the inoculum titre, and the inoculation route. Here, the higher mortality rates and earlier onset of clinical signs in the H5N8/2017 inoculated and contact groups than in the H5N8/2020 inoculated and contact groups suggest greater virulence and/or adaptation of H5N8/2017 in mule ducks. The difference in mortality rates between H5N8/2017-inoculated ducks and contact ducks could be attributed to differences in the infectious dose they received. In fact, following inoculation, all inoculated ducks started to shed viruses at a high rate, which likely contaminated the contact ducks with a higher infectious dose, leading to more severe infection and, consequently, a higher mortality rate.

Here, viral shedding was monitored for 14 days using officially recognized OP and CL swab samples [17] and additional nonofficial samples such as FP and CJ swabs. On the one hand, FP sampling was performed because early and high levels of vRNA are often detected in H5Nx HPAIV-infected ducks [30–32]. On the other hand, CJ swabs were included because HPAIV can replicate in the ocular system, including the conjunctival mucosa, cornea, and Harderian glands, of ducks, turkeys and chickens but not exclusively [27, 33–37]. Additionally, CJ swabs proved to be a reliable sample for Gs/GD clade 2.3.2.1 [38, 39] and clade 2.3.4.4 [40] HPAIV detection. In our study, all samples were already positive at 2 dpi, and in contrast to the onset of clinical signs, no difference in viral shedding between the two viral strains was detected, allowing up to two more days of H5N8/2020 presymptomatic viral shedding. Importantly, vRNA detection in contact bird samples suggested that bird-to-bird transmission begins during the first 24 h after the first viral exposure. Our findings confirmed previous observations of efficient presymptomatic viral shedding in ducks infected with Gs/GD HPAIVs, which differs from findings in other bird species, such as chickens, turkeys, ostriches, sparrows, crows or pheasants [11, 12, 21, 25, 27, 41–43]. Specifically, HPAIV-infected chickens typically have a short presymptomatic viral shedding period, and the onset of clinical signs is closely associated with rapid death, usually < 3 days [11, 12, 21, 25, 44]. Beerens et al. showed that chickens inoculated with different clade 2.3.4.4a and clade 2.3.4.4b H5Nx HPAIVs had much shorter OP shedding durations than did Pekin ducks (mean duration of 1.1–1.7 days in chickens vs 6.3–12.0 days in ducks) [12].

Analysis of unconventional CJ swabs and FP samples provided interesting results. Both sampling methods, such as OP and CL swabs, yielded early vRNA detection and high vRNA quantities. Interestingly, towards the end of the infection (10 and 14 dpi), both CJ swabs and FP samples showed high detection performance compared to CL swabs. Therefore, the use of CJ and FP samples could be a useful sampling strategy for viral detection in the field; although CJ swabbing requires trained personnel, similar to OP and CL swabbing, FP sampling is easy to perform, even by non-trained staff, and could constitute a valid alternative to CL and OP swabs.

To investigate the role of the environment in HPAIV transmission and to confirm previous findings regarding the potential of dust sampling for early detection and surveillance of HPAIV in farms [13], aerosols, dust, and water samples were collected throughout the experiment. The detection of vRNA in all environmental samples was in accordance with the viral shedding results. All environmental samples were positive for vRNA during the presymptomatic period (from 2 to 3 dpi in H5N8/2017 and from 2 to 5 dpi in H5N8/2020). Interestingly, H5N8/2020 drinking water and aerosols yielded positive results as early as 1 dpi, in line with the greater viral shedding observed in the H5N8/2020 group for the first 2 days than in the H5N8/2017 group, suggesting that these samples could be used for early viral detection in the field. Successful isolation of H5N8/2020 from drinking water at 1 dpi suggested high virus titres due to early host replication. Virus isolation was successful in all four types of environmental samples (drinking water, pool water, aerosols, and dust), but differences based on viral strain and sampling day were observed. In general, virus isolation was more successful on H5N8/2020 samples and around peak viral shedding days. Successful H5N8/2020 vRNA detection and viral isolation from both biological and environmental samples at 1 and 2 dpi confirmed that despite a longer incubation period, H5N8/2020-infected ducks shed more virus than H5N8/2017-infected ducks during the earliest days of infection. Importantly, virus isolation from environmental samples is difficult because all types of samples are subjected to a wide variety of stresses (e.g., chemical or physical) and contaminants that affect the successful isolation rate. The stresses induced in environmental samples and the presence of contaminants are not always easy to determine or quantify, hampering any correlation between RNA load and viral isolation. Quality control and standard analytical methods could play a major role in using environmental sampling for virus surveillance.

Our study compared a “stagnant water” model (pool water, not changed throughout the experiment) with a “renewed water” model (drinking water, daily changed). The stability of vRNA in pool water samples from 2–3 dpi up to 14 dpi could be explained by physical and chemical water parameters not limited to temperature, pH, or salinity [45, 46]. In contrast to those in pool water, vRNA in drinking water was not stable over time but rather mirrored the overall viral shedding kinetics observed in biological samples and other environmental samples. The role of water in HPAIV transmission has been widely investigated in recent years [10, 12, 43, 47–51], and waterborne infection in different bird species has been experimentally proven [10, 12, 49, 51]. Importantly, the transmission role of water among waterfowl could also be enhanced by animal behaviour, specifically by preening activities [52]. This finding offers new possibilities for the surveillance and early vRNA detection of HPAIV.

In contrast to drinking water, which is more likely to enable bird-to-bird transmission within commercial flocks, dust and aerosols can spread the virus at a larger scale. Our results confirm that airborne transmission of the infectious virus may lead to infection in contact birds; vRNA detection and virus isolation results for dust and aerosol samples are in agreement with the findings of viral shedding in contact birds. Airborne transmission of H5Nx HPAIVs, including biologically generated aerosols and aerosolized dust infection, has been largely documented in recent years [11, 13, 53–57]. Ample evidence has confirmed bird-to-bird transmission due to airborne particles [53, 54], but farm-to-farm spread has been more difficult to investigate due to sampling challenges mostly associated with weather and sampling device sensitivity [11, 51, 55]. To the best of our knowledge, direct evidence of farm-to-farm airborne transmission has not been demonstrated and has only been suggested by modelling approaches [56, 58].

The diversity of environmental samples that tested positive for HPAIV early in the course of infection raises numerous questions and challenges regarding the control of future HPAIV epizootics. In addition to respiratory and digestive shedding routes, growing feathers of domestic ducks have been established as an alternative mechanism for viral diffusion via epithelial infection through viremia, active viral replication in the feather epithelium, and subsequent release of contaminated debris [59]. The high infectivity of these viruses, their potential resistance in the environment [60], and their ability to contaminate different environmental matrix types can drastically impact current biosecurity measures, not only during the productive life of birds but also during their movement and culling operations. Overall, our results show that clade 2.3.4.4b H5N8 HPAIVs are spread not only by living animals but also by the environment in which infected animals live, such as water or dust, which can be aerosolized and lead to long-range dissemination [57]. Therefore, culling operations, as well as cleaning and disinfection, could pose a risk for further viral dissemination if not performed properly.

Efficient viral shedding during the presymptomatic period in H5N8 clade 2.3.4.4b HPAIV-experimentally infected mule ducks suggests that a passive detection strategy based on overt clinical signs is not optimal for containing viral spread. Viral RNA detection in environmental samples in the absence of clinical signs would allow for a quicker response, limiting the number of infected birds and the number of infectious particles shed. Environmental sampling, particularly drinking water and dust sampling, could be a valuable, easy-to-perform, fast, non-invasive, cheap, and accurate strategy for active HPAIV detection and surveillance activities on farms.

### Supplementary Information

**Additional file 1. Cycle threshold (Ct) values obtained by RT-qPCR from biological samples. **Summary table of all the cycle threshold (Ct) values obtained by RT-qPCR on all biological samples (cloacal swabs, oropharyngeal swabs, conjunctival swabs, and feather pulp) collected from H5N8/2017 and H5N8/2020 inoculated and contact birds.

**Additional file 2. Serology of H5N8/2017 and H5N8/2020 experimentally infected ducks. **Summary table of the ELISA and HI titres of blood samples collected pre- and post-inoculation from all birds.

**Additional file 3. Cycle threshold (Ct) values obtained by RT-qPCR from environmental samples from the H5N8/2017 experimentally infected group.** Summary table of the cycle threshold (Ct) values obtained by RT-qPCR from the environmental samples (aerosol NIOSH fraction 1, aerosol NIOSH fraction 2, aerosol NIOSH fraction 3, aerosol Coriolis, dust walls, dust feeders, pool water, and drinking water) collected from the H5N8/2017 experimentally infected group.

**Additional file 4. Cycle threshold (Ct) values obtained by RT-qPCR from environmental samples from the H5N8/2020 experimentally infected group. **Summary table of the cycle threshold (Ct) values obtained by RT-qPCR from the environmental samples (aerosol NIOSH fraction 1, aerosol NIOSH fraction 2, aerosol NIOSH fraction 3, aerosol Coriolis, dust walls, dust feeders, pool water, and drinking water) collected from the H5N8/2020 experimentally infected group.

## Data Availability

The datasets supporting the conclusions of this article are included within the article and its additional files.
