# Supplementary material for: Viral shedding and environmental dispersion of two clade 2.3.4.4b H5 high pathogenicity avian influenza viruses in experimentally infected mule ducks: implications for environmental sampling
Source: Vet Res. 2024 Aug 12;55:100. doi: 10.1186/s13567-024-01357-z (PMC11318174; doi:10.1186/s13567-024-01357-z)
Supplement: Supplementary file 1 — Additional file 1. Cycle threshold (Ct) values obtained by RT-qPCR from biological samples. Summary table of all the cycle threshold (Ct) values obtained by RT-qPCR on all biological samples (cloacal swabs, oropharyngeal swabs, conjunctival swabs, and feather pulp) collected from H5N8/2017 and H5N8/2020 inoculated and contact birds. [file 13567_2024_1357_MOESM1_ESM.docx]

**Additional file 1: Cycle threshold (Ct) values obtained by RT-qPCR from biological samples.** CL: cloacal swabs, OP: oropharyngeal swabs, CJ: conjunctival swabs, FP: feather pulp, I: Inoculated birds, and C: contact birds.

|  |  |  | 2 days post inoculation | | | | 4 days post inoculation | | | | 7 days post inoculation | | | | 10 days post inoculation | | | | 14 days post inoculation | | | |
| --- | --- | --- | --- | --- | --- | --- | --- | --- | --- | --- | --- | --- | --- | --- | --- | --- | --- | --- | --- | --- | --- | --- |
| Bird ID | Inoculation or Contact bird | Virus | CL | OP | CJ | FP | CL | OP | CJ | FP | CL | OP | CJ | FP | CL | OP | CJ | FP | CL | OP | CJ | FP |
| S1 | I | H5N8/2017 | 34.53 | 25.32 | 26.44 | NA | 20.71 | 17.33 | 14.52 | 17.15 | 30.6 | 27.35 | 26.75 | 17.07 | 35.61 | 28.98 | 27.79 | NA | NA | NA | NA | NA |
| S2 | I | H5N8/2017 | NA | 28.11 | 28.91 | 32.59 | 28.32 | 19.92 | 19.01 | 17.33 | 32.7 | 26.88 | 27.91 | 21.91 | 33.21 | 33.75 | 34.76 | 29.95 | NA | 30.23 | 35.29 | NA |
| S3 | I | H5N8/2017 | 30.42 | 19.48 | 21.45 | NA | 26.23 | 21.38 | 19.79 | 19.01 | 30.03 | 30.36 | 33.36 | 20.2 | NA | 33.13 | 36.00 | 25.55 | NA | NA | 34.38 | NA |
| S4 | I | H5N8/2017 | 28.38 | 21.41 | 18.17 | 20.81 | 21.02 | 20.23 | 18.54 | 14.14 | 31.27 | 32.15 | 29.68 | 19.3 | 35.72 | 35.60 | 35.84 | 28.41 | NA | NA | 35.14 | NA |
| S52 | I | H5N8/2017 | 33.76 | 32.29 | 34.3 | NA | 26.43 | 20.42 | 20.9 | 15.27 | 26.65 | 24.36 | 26.33 | 15.46 | NA | NA | NA | NA | NA | NA | NA | NA |
| S6 | I | H5N8/2017 | NA | 33.3 | NA | NA | 28.42 | 16.96 | 18.35 | 13.19 | 26.3 | 22.02 | 18.53 | 13.03 | NA | NA | NA | NA | NA | NA | NA | NA |
| S7 | I | H5N8/2017 | 33.92 | 25.49 | 26.13 | 29.55 | 13.5 | 17.54 | 14.99 | 33.47 | 27.69 | 28.64 | 25.62 | 32.87 | 35.57 | 35.52 | 30.10 | 30.99 | NA | NA | NA | 34.53 |
| S8 | I | H5N8/2017 | NA | 26.07 | 29.34 | NA | 25.56 | 22.66 | 16.91 | 19.25 | NA | 27.86 | 26.14 | 17.1 | 30.90 | 33.11 | 34.82 | 23.35 | NA | 34.89 | 36.52 | NA |
| S9 | I | H5N8/2017 | 31.75 | 19.96 | 16.52 | 19.91 | 27.78 | 18.07 | 16.38 | 12.01 | NA | NA | NA | NA | NA | NA | NA | NA | NA | NA | NA | NA |
| S10 | I | H5N8/2017 | 33.3 | 22.39 | 25.07 | NA | 25.39 | 20.64 | 17.72 | 15.72 | 28.8 | NA | 30.98 | 14.02 | NA | 35.23 | 33.50 | 22.73 | NA | NA | 34.37 | NA |
| S23 | I | H5N8/2021 | 26.05 | 20.91 | 17.9 | 33.84 | 31.43 | 23.36 | 22.99 | 21.02 | 34.97 | 32.12 | 32.45 | 35.15 | NA | NA | 31.66 | 28.00 | NA | NA | NA | NA |
| S24 | I | H5N8/2021 | 30.57 | 29.86 | 25.9 | 34.33 | 29.25 | 23.55 | 19.88 | 32.03 | 29.64 | 34.46 | 32.93 | NA | NA | 30.73 | NA | NA | NA | 36.12 | NA | 34.63 |
| S25 | I | H5N8/2021 | 30.99 | 23.93 | 19.16 | 25.56 | 30.9 | 21.09 | 22.03 | 18.66 | NA | NA | NA | NA | NA | NA | NA | NA | NA | NA | NA | NA |
| S26 | I | H5N8/2021 | 26.77 | 20.6 | 19.87 | 19.33 | 30.87 | 23.24 | 20.41 | 16.29 | 29.92 | NA | 34.85 | 26.54 | NA | NA | 33.59 | NA | NA | 34.21 | NA | NA |
| S27 | I | H5N8/2021 | 34.23 | 27.03 | 20.88 | NA | 29.48 | 23.34 | 18.07 | 19.45 | 29.85 | NA | 33.17 | 29.01 | 36.47 | NA | 35.09 | 35.50 | NA | 35.15 | 35.93 | NA |
| S28 | I | H5N8/2021 | 26.71 | 22.82 | 18.06 | 20.71 | 26.84 | 21.53 | 19.66 | 29.9 | 31.01 | 30.11 | 32.93 | 26.23 | NA | NA | 34.02 | NA | NA | 29.06 | NA | NA |
| S29 | I | H5N8/2021 | 32.15 | 23.31 | 18.17 | 21.15 | 31.21 | 22.07 | 21.51 | 33.08 | 34.41 | 34.02 | 35.68 | 29.18 | NA | NA | 34.91 | NA | NA | 34.09 | NA | NA |
| S30 | I | H5N8/2021 | 32.92 | 27.11 | 23.27 | 29.91 | 30.45 | 28.57 | 26.5 | NA | 29.27 | 33.9 | 33.11 | NA | NA | NA | 33.59 | 35.83 | NA | NA | NA | NA |
| S31 | I | H5N8/2021 | NA | 20.24 | 17.32 | 35.4 | 32.54 | 21.06 | 19.96 | 19.46 | NA | NA | 32.3 | 25.06 | NA | NA | 32.66 | 29.55 | 35.90 | 34.46 | NA | 33.75 |
| S32 | I | H5N8/2021 | 25.41 | 21.11 | 22.66 | 18.44 | 31.65 | 22.12 | 22.13 | 16.7 | NA | NA | NA | NA | NA | NA | NA | NA | NA | NA | NA | NA |
| S11 | C | H5N8/2017 | 33.36 | 32.32 | NA | NA | 25.15 | 18.57 | 18.53 | 11.69 | NA | NA | NA | NA | NA | NA | NA | NA | NA | NA | NA | NA |
| S12 | C | H5N8/2017 | 33.76 | 34.67 | 35.18 | NA | 25.57 | 17.94 | 18.41 | 16.63 | NA | NA | NA | NA | NA | NA | NA | NA | NA | NA | NA | NA |
| S13 | C | H5N8/2017 | NA | 35.25 | 34.8 | NA | 25.62 | 17.9 | 17.23 | 18.15 | 30.7 | 19.31 | 18.56 | 33.61 | NA | NA | NA | NA | NA | NA | NA | NA |
| S14 | C | H5N8/2017 | NA | 34.46 | NA | NA | 25.85 | 19.88 | 19.98 | 33.87 | 26.9 | 25.59 | 24.49 | 15.16 | NA | 32.59 | 31.87 | 27.94 | NA | NA | NA | NA |
| S15 | C | H5N8/2017 | 33.08 | 33.47 | 33.4 | NA | 23.38 | 19.49 | 17.07 | 14.05 | NA | NA | NA | NA | NA | NA | NA | NA | NA | NA | NA | NA |
| S16 | C | H5N8/2017 | 33.35 | 33.5 | 34.26 | NA | 29.32 | 18.86 | 19.41 | 26.48 | 30.28 | 25.65 | 24.29 | 15.17 | NA | NA | NA | NA | NA | NA | NA | NA |
| S33 | C | H5N8/2021 | NA | 33.44 | 35.05 | NA | 30.98 | 19.79 | 19.56 | 18.18 | 34.78 | 31.21 | 27.57 | 31.42 | 35.08 | 32.99 | 34.87 | 29.44 | NA | NA | NA | NA |
| S34 | C | H5N8/2021 | 32.23 | 32.86 | 32.18 | NA | 29.2 | 20.25 | 19.11 | 19.94 | NA | 30.09 | 29.16 | 28.87 | NA | NA | NA | NA | NA | 36.01 | NA | 33.54 |
| S35 | C | H5N8/2021 | 33.69 | 31.47 | 28.71 | NA | 31.25 | 21.14 | 19.43 | 30.43 | NA | 28.46 | 26.86 | 28.36 | NA | NA | 32.85 | NA | NA | NA | NA | 33.63 |
| S36 | C | H5N8/2021 | NA | 33.76 | 32.02 | 35.51 | 31.33 | 20.78 | 19.91 | 16.53 | 33.21 | 33.97 | 30.38 | 22.33 | 32.93 | NA | NA | NA | NA | NA | 34.33 | 34.30 |
| S37 | C | H5N8/2021 | 35.73 | 34.32 | 33.54 | 34.27 | 28.31 | 18.99 | 18.38 | 19.32 | NA | 26.25 | 29.36 | 16.56 | NA | NA | NA | NA | NA | NA | NA | NA |
| S38 | C | H5N8/2021 | 30.74 | 31.85 | 35.01 | NA | 31.39 | 21.53 | 20.64 | 20.79 | NA | 28.03 | 29.75 | 35.51 | NA | 35.30 | 36.00 | NA | NA | NA | NA | 29.44 |
