# Supplementary material for: Viral shedding and environmental dispersion of two clade 2.3.4.4b H5 high pathogenicity avian influenza viruses in experimentally infected mule ducks: implications for environmental sampling
Source: Vet Res. 2024 Aug 12;55:100. doi: 10.1186/s13567-024-01357-z (PMC11318174; doi:10.1186/s13567-024-01357-z)
Supplement: Supplementary file 2 — Additional file 2. Serology of H5N8/2017 and H5N8/2020 experimentally infected ducks. Summary table of the ELISA and HI titres of blood samples collected pre- and post-inoculation from all birds. [file 13567_2024_1357_MOESM2_ESM.docx]

**Additional file 2:** **Serology of ducks experimentally-infected with A/mulard duck/France/171201g/2017 (H5N8) HPAIV (H5N8/2017) and A/Mule_duck/France/20353/2020 (H5N8/2020).** ELISA and HI antibody titers (expressed as log_2_ GMT) against corresponding challenge viruses in pre-inoculation and post-inoculation (time of death) sera. Neg: negative. Pos: positive. GMT: geometrical mean titers.

|  |  | **Pre-inoculation** | | **Post-inoculation** | | |  |
| --- | --- | --- | --- | --- | --- | --- | --- |
|  | **Animal ID** | **ELISA** | **HI (antigen H5N8)** | **day of death/euthanasia** | **ELISA** | **HI (antigen H5N8)** | **GMT** |
| **H5N8/2017** | **S1** | neg | neg | 14 | pos | 8 | 8.4 |
|  | **S2** | neg | neg | 14 | pos | 9 |  |
|  | **S3** | neg | neg | 14 | pos | 9 |  |
|  | **S4** | neg | neg | 14 | pos | 8 |  |
|  | **S52** | neg | neg | 8 | pos | neg |  |
|  | **S6** | neg | neg | 7 | pos | 2 |  |
|  | **S7** | neg | neg | 14 | pos | 9 |  |
|  | **S8** | neg | neg | 14 | pos | 9 |  |
|  | **S9** | neg | neg | 5 | pos | neg |  |
|  | **S10** | neg | neg | 14 | pos | 7 |  |
|  | **S11** | neg | neg | 6 | pos | neg |  |
|  | **S12** | neg | neg | 6 | pos | neg |  |
|  | **S13** | neg | neg | 7 | pos | neg |  |
|  | **S14** | neg | neg | 11 | pos | 6 |  |
|  | **S15** | neg | neg | 6 | pos | neg |  |
|  | **S16** | neg | neg | 8 | pos | neg |  |
| **H5N8/2020** | **S23** | neg | neg | 14 | pos | 10 | 8.6 |
|  | **S24** | neg | neg | 14 | pos | 8 |  |
|  | **S25** | neg | neg | 6 | pos | 3 |  |
|  | **S26** | neg | neg | 14 | pos | 10 |  |
|  | **S27** | neg | neg | 14 | pos | 9 |  |
|  | **S28** | neg | neg | 14 | pos | 7 |  |
|  | **S29** | neg | neg | 14 | pos | 8 |  |
|  | **S30** | neg | neg | 14 | pos | 8 |  |
|  | **S31** | neg | neg | 14 | pos | 9 |  |
|  | **S32** | neg | neg | 5 | pos | neg |  |
|  | **S33** | neg | neg | 11 | pos | 6 | 7.5 |
|  | **S34** | neg | neg | 14 | pos | 8 |  |
|  | **S35** | neg | neg | 14 | pos | 7 |  |
|  | **S36** | neg | neg | 14 | pos | 7 |  |
|  | **S37** | neg | neg | 7 | pos | neg |  |
|  | **S38** | neg | neg | 14 | pos | 8 |  |
