# Supplementary material for: Viral shedding and environmental dispersion of two clade 2.3.4.4b H5 high pathogenicity avian influenza viruses in experimentally infected mule ducks: implications for environmental sampling
Source: Vet Res. 2024 Aug 12;55:100. doi: 10.1186/s13567-024-01357-z (PMC11318174; doi:10.1186/s13567-024-01357-z)
Supplement: Supplementary file 3 — Additional file 3. Cycle threshold (Ct) values obtained by RT-qPCR from environmental samples from the H5N8/2017 experimentally infected group. Summary table of the cycle threshold (Ct) values obtained by RT-qPCR from the environmental samples (aerosol NIOSH fraction 1, aerosol NIOSH fraction 2, aerosol NIOSH fraction 3, aerosol Coriolis, dust walls, dust feeders, pool water, and drinking water) collected from the H5N8/2017 experimentally infected group. [file 13567_2024_1357_MOESM3_ESM.docx]

**Additional file 3** **Cycle threshold (Ct) values obtained by RT-qPCR from environmental samples from the H5N8/2017 group**. dpi: days post-inoculation. NIOSH fraction 1 <4µm, NIOSH fraction 2 1-to-4µm, NIOSH fraction 3 <1µm.

| **H5N8/2017** | **Sampling day** | | | | | | | |
| --- | --- | --- | --- | --- | --- | --- | --- | --- |
| Sample type | 1 dpi | 2 dpi | 3 dpi | 4 dpi | 5 dpi | 7 dpi | 10 dpi | 14 dpi |
| Aerosols NIOSH Fraction 1 | Neg | Neg | 26.6 | Neg | 23.59 | 29.28 | 33.31 | 31.96 |
| Aerosols NIOSH Fraction 2 | Neg | Neg | Neg | 31.23 | 29.66 | 35.71 | Neg | Neg |
| Aerosols NIOSH Fraction 3 | Neg | Neg | Neg | Neg | Neg | 34.99 | Neg | Neg |
| Aerosols Coriolis | Neg | 33.9 | 22.31 | 22.65 | 19.74 | 25.08 | 29.99 | 29.78 |
| Dust walls | Neg | 27.12 | 28.06 | 22.03 | 20.47 | 24.63 | 20.71 | 29.91 |
| Dust feeders | Neg | 31.98 | 25.54 | 23.04 | 21.44 | 26.82 | 28.07 | 24.81 |
| Pool water | Neg | 32.11 | 26.89 | 26.58 | 26.45 | 26.86 | 26.48 | 28.18 |
| Drinking water | Neg | 28.81 | 22.78 | 21.26 | 18.86 | 22.46 | 30.55 | 32.89 |
